# 2-(4-Meth­oxy­phen­yl)-4-oxo-4-phenyl­butane­nitrile

**DOI:** 10.1107/S1600536812006290

**Published:** 2012-02-17

**Authors:** Alaa A.-M. Abdel-Aziz, Adel S. El-Azab, Seik Weng Ng, Edward R. T. Tiekink

**Affiliations:** a Department of Pharmaceutical Chemistry, College of Pharmacy, King Saud University, Riyadh 11451, Saudi Arabia; b Department of Medicinal Chemistry, Faculty of Pharmacy, University of Mansoura, Mansoura 35516, Egypt; c Department of Organic Chemistry, Faculty of Pharmacy, Al-Azhar University, Cairo 11884, Egypt; d Department of Chemistry, University of Malaya, 50603 Kuala Lumpur, Malaysia; e Chemistry Department, Faculty of Science, King Abdulaziz University, PO Box 80203 Jeddah, Saudi Arabia

## Abstract

The title mol­ecule, C_17_H_15_NO_2_, is twisted, the dihedral angle between the terminal benzene rings being 63.30 (6)°. In the crystal, C—H⋯O and C—H⋯N inter­actions lead to supra­molecular layers in the *ab* plane. These are connected along the *c* axis *via* C—H⋯π inter­actions.

## Related literature

For background to the synthetic applications of 2,4-diaryl-4-oxo-butane­nitriles, see: Coudert *et al.* (1990 ▶), 1988 ▶); Iida *et al.* (2007 ▶). For the preparation of the title compound, see: Coudert *et al.* (1990 ▶). For the structure of the unsubstituted parent compound, see: Abdel-Aziz *et al.* (2012 ▶).
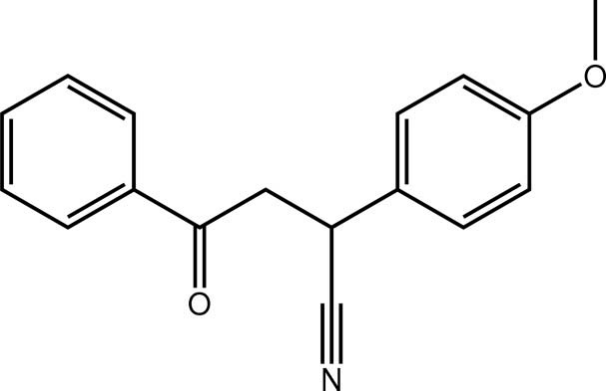


## Experimental

### 

#### Crystal data

C_17_H_15_NO_2_
*M* *_r_* = 265.30
Orthorhombic, 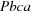

*a* = 9.5730 (2) Å
*b* = 8.7748 (2) Å
*c* = 32.0620 (7) Å
*V* = 2693.25 (10) Å^3^
*Z* = 8
Cu *K*α radiation
μ = 0.69 mm^−1^
*T* = 100 K
0.30 × 0.30 × 0.05 mm

#### Data collection

Agilent SuperNova Dual diffractometer with an Atlas detector
Absorption correction: multi-scan (*CrysAlis PRO*; Agilent, 2011 ▶) *T*_min_ = 0.651, *T*_max_ = 1.000
6569 measured reflections
2764 independent reflections
2410 reflections with *I* > 2σ(*I*)
*R*_int_ = 0.019

#### Refinement

*R*[*F* ^2^ > 2σ(*F* ^2^)] = 0.037
*wR*(*F* ^2^) = 0.101
*S* = 1.02
2764 reflections
181 parameters
H-atom parameters constrained
Δρ_max_ = 0.21 e Å^−3^
Δρ_min_ = −0.21 e Å^−3^

### 

Data collection: *CrysAlis PRO* (Agilent, 2011 ▶); cell refinement: *CrysAlis PRO*; data reduction: *CrysAlis PRO*; program(s) used to solve structure: *SHELXS97* (Sheldrick, 2008 ▶); program(s) used to refine structure: *SHELXL97* (Sheldrick, 2008 ▶); molecular graphics: *ORTEP-3* (Farrugia, 1997 ▶) and *DIAMOND* (Brandenburg, 2006 ▶); software used to prepare material for publication: *publCIF* (Westrip, 2010 ▶).

## Supplementary Material

Crystal structure: contains datablock(s) global, I. DOI: 10.1107/S1600536812006290/xu5470sup1.cif

Structure factors: contains datablock(s) I. DOI: 10.1107/S1600536812006290/xu5470Isup2.hkl

Supplementary material file. DOI: 10.1107/S1600536812006290/xu5470Isup3.cml

Additional supplementary materials:  crystallographic information; 3D view; checkCIF report

## Figures and Tables

**Table 1:** Hydrogen-bond geometry (Å, °) *Cg* is the centroid of the C11–C16 ring.

*D*—H⋯*A*	*D*—H	H⋯*A*	*D*⋯*A*	*D*—H⋯*A*
C8—H8b⋯O1^i^	0.99	2.44	3.3102 (16)	147
C15—H15⋯N1^ii^	0.95	2.62	3.4250 (17)	143
C4—H4⋯*Cg*^iii^	0.95	2.82	3.5787 (14)	138
C17—H17c⋯*Cg*^iv^	0.98	2.89	3.6754 (15)	138

Symmetry codes: (i) 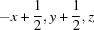; (ii) 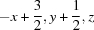; (iii) 
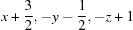
; (iv) 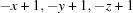.

**Fig. 1.**
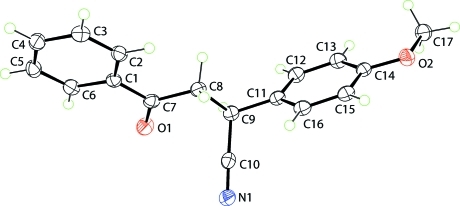
The molecular structure of (I) showing the atom-labelling scheme and displacement ellipsoids at the 50% probability level.

**Fig. 2.**
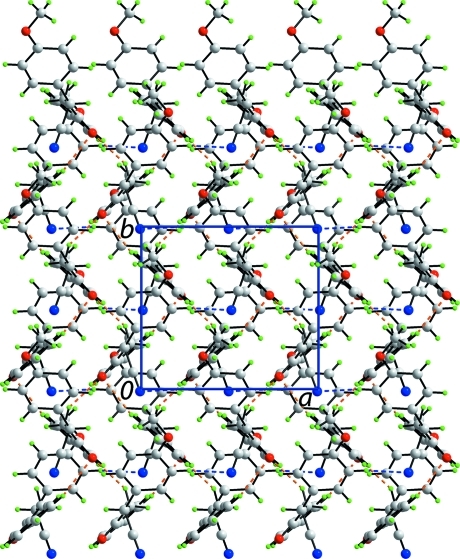
A view of the supramolecular array in the ab plane in (I). The C—H···O and C—H···N interactions are shown as orange and blue dashed lines, respectively.

**Fig. 3.**
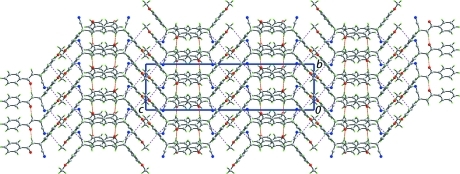
A view in projection down the a axis of the unit-cell contents for (I). The C—H···O, C—H···N and C—H···π interactions are shown as orange, blue and purple dashed lines, respectively.

## supplementary crystallographic information

### Comment

An important class of difunctional intermediates for both the synthesis of biologically active heterocycles, such as pyridazine derivatives, and as a source ketone (Coudert *et al.*, 1990; Coudert *et al.*, 1988; Iida *et al.*, 2007) are the 2,4-diaryl-4-oxo-butanenitriles. Herein, the crystal structure of a 2,4-diaryl-4-oxo-butanenitrile derivative, 4-(4-methoxyphenyl)-4-oxo-2-phenylbutanenitrile (I), is described. The structure of the parent compound is known (Abdel-Aziz *et al.*, 2012).

In (I), Fig. 1, the terminal benzene rings form a dihedral angle of 63.30 (6)° indicating a considerable twist in the molecule. The benzyl group is twisted out of the plane of the benzene ring to which it is connected [the C2—C1—C7—C8 torsion angle is -8.58 (17)°] and in addition there is a twist around the C8—C9 bond [the C7—C8—C9—C11 torsion angle is 173.19 (10)°]. The methoxy group is co-planar with the benzene ring to which it is connected [the C17—O2—C14—C13 torsion angle is 3.03 (17)°].

In the crystal packing, molecules are linked by C—H···O and C—H···N interactions into supramolecular layers in the *ab* plane, Fig. 2 and Table 1. Layers are connected along the *c* axis *via* C—H···π interactions involving the (C11–C16) ring, Fig. 3 and Table 1.

### Experimental

Acetone cyanohydrin (0.045 mol) and 10% aqueous sodium carbonate (0.0015 mol and 1.5 ml water) were added to solution of 3-(4-methoxyphenyl)-1-phenylprop-2-en-1-one (0.015 mol) in ethanol (50 ml). The mixture was heated at reflux temperature for 4 h. After cooling, the product which separated out was filtered off and recrystallized from methanol solution.

### Refinement

Carbon-bound H-atoms were placed in calculated positions [C—H = 0.95 to 1.00 Å, *U*_iso_(H) = 1.2–1.5*U*_eq_(C)] and were included in the refinement in the riding model approximation.

### Crystal data

**Table:**

C_17_H_15_NO_2_	*F*(000) = 1120
*M**_r_* = 265.30	*D*_x_ = 1.309 Mg m^−^^3^
Orthorhombic, *P**b**c**a*	Cu *K*α radiation, λ = 1.5418 Å
Hall symbol: -P 2ac 2ab	Cell parameters from 2587 reflections
*a* = 9.5730 (2) Å	θ = 2.8–76.0°
*b* = 8.7748 (2) Å	µ = 0.69 mm^−^^1^
*c* = 32.0620 (7) Å	*T* = 100 K
*V* = 2693.25 (10) Å^3^	Prism, colourless
*Z* = 8	0.30 × 0.30 × 0.05 mm

### Data collection

**Table:**

Agilent SuperNova Dual diffractometer with an Atlas detector	2764 independent reflections
Radiation source: SuperNova (Cu) X-ray Source	2410 reflections with *I* > 2σ(*I*)
Mirror monochromator	*R*_int_ = 0.019
Detector resolution: 10.4041 pixels mm^-1^	θ_max_ = 76.2°, θ_min_ = 2.8°
ω scan	*h* = −11→11
Absorption correction: multi-scan (*CrysAlis PRO*; Agilent, 2011)	*k* = −10→10
*T*_min_ = 0.651, *T*_max_ = 1.000	*l* = −33→39
6569 measured reflections	

### Refinement

**Table:**

Refinement on *F*^2^	Primary atom site location: structure-invariant direct methods
Least-squares matrix: full	Secondary atom site location: difference Fourier map
*R*[*F*^2^ > 2σ(*F*^2^)] = 0.037	Hydrogen site location: inferred from neighbouring sites
*wR*(*F*^2^) = 0.101	H-atom parameters constrained
*S* = 1.02	*w* = 1/[σ^2^(*F*_o_^2^) + (0.0514*P*)^2^ + 0.8432*P*] where *P* = (*F*_o_^2^ + 2*F*_c_^2^)/3
2764 reflections	(Δ/σ)_max_ = 0.001
181 parameters	Δρ_max_ = 0.21 e Å^−^^3^
0 restraints	Δρ_min_ = −0.21 e Å^−^^3^

### Special details

**Table:**

Geometry. All e.s.d.'s (except the e.s.d. in the dihedral angle between two l.s. planes) are estimated using the full covariance matrix. The cell e.s.d.'s are taken into account individually in the estimation of e.s.d.'s in distances, angles and torsion angles; correlations between e.s.d.'s in cell parameters are only used when they are defined by crystal symmetry. An approximate (isotropic) treatment of cell e.s.d.'s is used for estimating e.s.d.'s involving l.s. planes.
Refinement. Refinement of *F*^2^ against ALL reflections. The weighted *R*-factor *wR* and goodness of fit *S* are based on *F*^2^, conventional *R*-factors *R* are based on *F*, with *F* set to zero for negative *F*^2^. The threshold expression of *F*^2^ > σ(*F*^2^) is used only for calculating *R*-factors(gt) *etc*. and is not relevant to the choice of reflections for refinement. *R*-factors based on *F*^2^ are statistically about twice as large as those based on *F*, and *R*- factors based on ALL data will be even larger.

### Fractional atomic coordinates and isotropic or equivalent isotropic displacement parameters (Å^2^)

**Table:**

	*x*	*y*	*z*	*U*_iso_*/*U*_eq_	
O1	0.27711 (10)	0.07603 (11)	0.68494 (3)	0.0294 (2)	
O2	0.65190 (10)	0.70231 (10)	0.52150 (3)	0.0267 (2)	
N1	0.49228 (12)	−0.01483 (13)	0.60258 (3)	0.0282 (3)	
C1	0.37272 (13)	0.18133 (13)	0.74651 (4)	0.0214 (3)	
C2	0.47344 (13)	0.27465 (14)	0.76484 (4)	0.0235 (3)	
H2	0.5322	0.3358	0.7478	0.028*	
C3	0.48802 (14)	0.27836 (15)	0.80803 (4)	0.0261 (3)	
H3	0.5570	0.3418	0.8204	0.031*	
C4	0.40215 (14)	0.18967 (15)	0.83309 (4)	0.0262 (3)	
H4	0.4124	0.1922	0.8625	0.031*	
C5	0.30090 (14)	0.09693 (15)	0.81498 (4)	0.0268 (3)	
H5	0.2420	0.0364	0.8321	0.032*	
C6	0.28593 (13)	0.09281 (15)	0.77198 (4)	0.0244 (3)	
H6	0.2165	0.0297	0.7598	0.029*	
C7	0.35473 (13)	0.17095 (14)	0.70034 (4)	0.0225 (3)	
C8	0.43447 (13)	0.28103 (14)	0.67269 (4)	0.0231 (3)	
H8A	0.5359	0.2687	0.6777	0.028*	
H8B	0.4086	0.3868	0.6802	0.028*	
C9	0.40397 (13)	0.25499 (14)	0.62604 (4)	0.0228 (3)	
H9	0.3005	0.2589	0.6220	0.027*	
C10	0.45287 (13)	0.10206 (15)	0.61327 (4)	0.0230 (3)	
C11	0.46935 (13)	0.37597 (14)	0.59828 (4)	0.0222 (3)	
C12	0.38502 (13)	0.48068 (15)	0.57791 (4)	0.0251 (3)	
H12	0.2867	0.4759	0.5816	0.030*	
C13	0.44116 (14)	0.59264 (15)	0.55215 (4)	0.0252 (3)	
H13	0.3818	0.6640	0.5386	0.030*	
C14	0.58517 (14)	0.59909 (14)	0.54638 (4)	0.0219 (3)	
C15	0.67095 (13)	0.49409 (14)	0.56660 (4)	0.0230 (3)	
H15	0.7693	0.4985	0.5628	0.028*	
C16	0.61369 (13)	0.38362 (14)	0.59212 (4)	0.0233 (3)	
H16	0.6730	0.3122	0.6056	0.028*	
C17	0.56675 (16)	0.81515 (16)	0.50178 (4)	0.0293 (3)	
H17A	0.6249	0.8788	0.4836	0.044*	
H17B	0.5226	0.8790	0.5231	0.044*	
H17C	0.4944	0.7650	0.4851	0.044*	

### Atomic displacement parameters (Å^2^)

**Table:**

	*U*^11^	*U*^22^	*U*^33^	*U*^12^	*U*^13^	*U*^23^
O1	0.0261 (5)	0.0329 (5)	0.0292 (5)	−0.0061 (4)	0.0019 (4)	−0.0041 (4)
O2	0.0286 (5)	0.0262 (5)	0.0254 (4)	0.0001 (4)	0.0000 (4)	0.0033 (4)
N1	0.0313 (6)	0.0268 (6)	0.0264 (5)	−0.0021 (5)	0.0035 (5)	−0.0028 (4)
C1	0.0201 (6)	0.0188 (6)	0.0252 (6)	0.0040 (5)	0.0029 (5)	0.0002 (4)
C2	0.0228 (6)	0.0201 (6)	0.0276 (6)	0.0005 (5)	0.0042 (5)	0.0007 (5)
C3	0.0250 (6)	0.0240 (6)	0.0294 (6)	0.0010 (5)	−0.0003 (5)	−0.0027 (5)
C4	0.0283 (6)	0.0264 (7)	0.0239 (6)	0.0055 (5)	0.0015 (5)	0.0021 (5)
C5	0.0247 (6)	0.0264 (6)	0.0294 (6)	0.0027 (5)	0.0066 (5)	0.0053 (5)
C6	0.0203 (6)	0.0220 (6)	0.0309 (6)	0.0003 (5)	0.0023 (5)	0.0004 (5)
C7	0.0188 (5)	0.0213 (6)	0.0274 (6)	0.0032 (5)	0.0024 (5)	−0.0028 (5)
C8	0.0235 (6)	0.0227 (6)	0.0232 (6)	0.0008 (5)	0.0013 (5)	−0.0022 (5)
C9	0.0204 (6)	0.0239 (6)	0.0241 (6)	0.0012 (5)	−0.0005 (5)	−0.0009 (5)
C10	0.0227 (6)	0.0268 (7)	0.0197 (5)	−0.0034 (5)	0.0005 (5)	−0.0003 (5)
C11	0.0234 (6)	0.0236 (6)	0.0197 (5)	0.0014 (5)	−0.0005 (5)	−0.0029 (5)
C12	0.0202 (6)	0.0281 (7)	0.0269 (6)	0.0029 (5)	−0.0003 (5)	−0.0013 (5)
C13	0.0255 (6)	0.0255 (6)	0.0246 (6)	0.0048 (5)	−0.0035 (5)	0.0000 (5)
C14	0.0263 (6)	0.0208 (6)	0.0186 (5)	−0.0005 (5)	−0.0001 (5)	−0.0028 (4)
C15	0.0193 (6)	0.0260 (6)	0.0237 (6)	0.0015 (5)	−0.0010 (5)	−0.0041 (5)
C16	0.0235 (6)	0.0238 (6)	0.0225 (6)	0.0043 (5)	−0.0032 (5)	−0.0023 (5)
C17	0.0375 (7)	0.0271 (7)	0.0233 (6)	0.0032 (6)	−0.0012 (5)	0.0035 (5)

### Geometric parameters (Å, º)

**Table:**

O1—C7	1.2205 (15)	C8—H8A	0.9900
O2—C14	1.3656 (15)	C8—H8B	0.9900
O2—C17	1.4299 (15)	C9—C10	1.4791 (17)
N1—C10	1.1454 (17)	C9—C11	1.5201 (17)
C1—C2	1.3949 (17)	C9—H9	1.0000
C1—C6	1.4002 (17)	C11—C12	1.3865 (17)
C1—C7	1.4931 (17)	C11—C16	1.3975 (18)
C2—C3	1.3921 (18)	C12—C13	1.3915 (18)
C2—H2	0.9500	C12—H12	0.9500
C3—C4	1.3881 (18)	C13—C14	1.3920 (18)
C3—H3	0.9500	C13—H13	0.9500
C4—C5	1.3924 (19)	C14—C15	1.3941 (17)
C4—H4	0.9500	C15—C16	1.3818 (18)
C5—C6	1.3865 (18)	C15—H15	0.9500
C5—H5	0.9500	C16—H16	0.9500
C6—H6	0.9500	C17—H17A	0.9800
C7—C8	1.5172 (17)	C17—H17B	0.9800
C8—C9	1.5409 (16)	C17—H17C	0.9800
			
C14—O2—C17	116.80 (10)	C11—C9—C8	112.75 (10)
C2—C1—C6	119.34 (11)	C10—C9—H9	108.0
C2—C1—C7	122.23 (11)	C11—C9—H9	108.0
C6—C1—C7	118.43 (11)	C8—C9—H9	108.0
C3—C2—C1	120.14 (12)	N1—C10—C9	178.38 (13)
C3—C2—H2	119.9	C12—C11—C16	118.50 (12)
C1—C2—H2	119.9	C12—C11—C9	119.93 (11)
C4—C3—C2	120.21 (12)	C16—C11—C9	121.57 (11)
C4—C3—H3	119.9	C11—C12—C13	121.51 (12)
C2—C3—H3	119.9	C11—C12—H12	119.2
C3—C4—C5	119.91 (12)	C13—C12—H12	119.2
C3—C4—H4	120.0	C14—C13—C12	119.34 (12)
C5—C4—H4	120.0	C14—C13—H13	120.3
C6—C5—C4	120.12 (12)	C12—C13—H13	120.3
C6—C5—H5	119.9	O2—C14—C13	124.60 (11)
C4—C5—H5	119.9	O2—C14—C15	115.75 (11)
C5—C6—C1	120.28 (12)	C13—C14—C15	119.65 (12)
C5—C6—H6	119.9	C16—C15—C14	120.35 (12)
C1—C6—H6	119.9	C16—C15—H15	119.8
O1—C7—C1	120.86 (11)	C14—C15—H15	119.8
O1—C7—C8	120.30 (11)	C15—C16—C11	120.64 (12)
C1—C7—C8	118.85 (11)	C15—C16—H16	119.7
C7—C8—C9	112.17 (10)	C11—C16—H16	119.7
C7—C8—H8A	109.2	O2—C17—H17A	109.5
C9—C8—H8A	109.2	O2—C17—H17B	109.5
C7—C8—H8B	109.2	H17A—C17—H17B	109.5
C9—C8—H8B	109.2	O2—C17—H17C	109.5
H8A—C8—H8B	107.9	H17A—C17—H17C	109.5
C10—C9—C11	109.95 (10)	H17B—C17—H17C	109.5
C10—C9—C8	110.08 (10)		
			
C6—C1—C2—C3	0.58 (18)	C10—C9—C11—C12	126.52 (12)
C7—C1—C2—C3	−178.79 (11)	C8—C9—C11—C12	−110.23 (13)
C1—C2—C3—C4	−0.23 (19)	C10—C9—C11—C16	−52.54 (15)
C2—C3—C4—C5	−0.13 (19)	C8—C9—C11—C16	70.70 (15)
C3—C4—C5—C6	0.13 (19)	C16—C11—C12—C13	−0.69 (18)
C4—C5—C6—C1	0.23 (19)	C9—C11—C12—C13	−179.78 (11)
C2—C1—C6—C5	−0.58 (18)	C11—C12—C13—C14	0.55 (19)
C7—C1—C6—C5	178.81 (11)	C17—O2—C14—C13	3.03 (17)
C2—C1—C7—O1	171.59 (11)	C17—O2—C14—C15	−177.49 (10)
C6—C1—C7—O1	−7.78 (17)	C12—C13—C14—O2	179.13 (11)
C2—C1—C7—C8	−8.58 (17)	C12—C13—C14—C15	−0.33 (18)
C6—C1—C7—C8	172.05 (11)	O2—C14—C15—C16	−179.24 (10)
O1—C7—C8—C9	−0.11 (16)	C13—C14—C15—C16	0.27 (18)
C1—C7—C8—C9	−179.94 (10)	C14—C15—C16—C11	−0.42 (18)
C7—C8—C9—C10	−63.64 (13)	C12—C11—C16—C15	0.62 (18)
C7—C8—C9—C11	173.19 (10)	C9—C11—C16—C15	179.70 (11)

### Hydrogen-bond geometry (Å, º)

Cg is the centroid of the C11–C16 ring.

**Table:**

*D*—H···*A*	*D*—H	H···*A*	*D*···*A*	*D*—H···*A*
C8—H8b···O1^i^	0.99	2.44	3.3102 (16)	147
C15—H15···N1^ii^	0.95	2.62	3.4250 (17)	143
C4—H4···*Cg*^iii^	0.95	2.82	3.5787 (14)	138
C17—H17c···*Cg*^iv^	0.98	2.89	3.6754 (15)	138

Symmetry codes: (i) −*x*+1/2, *y*+1/2, *z*; (ii) −*x*+3/2, *y*+1/2, *z*; (iii) *x*+3/2, −*y*−1/2, −*z*+1; (iv) −*x*+1, −*y*+1, −*z*+1.
